# In Memoriam: Louis J. Guillette, Jr.

**DOI:** 10.1289/ehp.1510674

**Published:** 2015-10-01

**Authors:** Caren C. Helbing, Charles R. Tyler, Taisen Iguchi

Louis (Lou) J. Guillette, Jr., one of the most influential research scientists in the field of environmental health, died 6 August 2015 at the age of 60. Lou had been battling non-Hodgkin’s lymphoma for almost a decade, but his passing, caused by bacterial pneumonia, came as a shock to everyone who knew him. He was an extremely energetic man with a passion for the natural environment. He revelled in being a scientist, adventurer, artist, and storyteller, and he was a talented communicator. Lou helped lead environmental research into a new era and is most recognized for identifying endocrine disruption in alligators and linking that groundbreaking research to consequences on human health. He was instrumental in instigating a paradigm shift in toxicology: the recognition that a change in hormone signaling during critical windows of development caused by low-dose exposure can lead to deleterious health effects, including cancer.

Lou was born at Sheppard Air Force Base in Wichita Falls, Texas, the oldest of four children. After completing his BSc degree at New Mexico Highlands University, he worked as a wildlife biologist observing wild turkey behavior in New Mexico. Interested in graduate school, he approached Dick Jones at the University of Colorado to join his lab. Dick recalls being impressed with Lou’s “remarkable and natural ability to form preliminary research questions” and his “unusual amount of excitement and enthusiasm to do the detective work of creative research in biology.” Lou completed an MA in 1979 and a PhD in 1981 with 10 publications. His graduate experience sparked one of his lifelong research passions—the evolution of the reproductive system, particularly sex determination, viviparity, and placentation—and set the groundwork for what was to become an exemplary career in research and teaching.

After a brief stint as an assistant professor at Wichita State University, Lou was recruited to the University of Florida, Gainesville, in 1985, where he set up his own laboratory of comparative endocrinology. In a partnership with the Florida Game and Fresh Water Fish Commission, Lou began his studies on the reproduction of the American alligator, for which he became world famous. Alligators had both commercial and conservation interest at the time, but little was known about their basic biology. Lou began characterizing the general health of alligators in nearby waters, and he discovered that some populations had significant reproductive problems. Systematically eliminating the possible causative factors led him to the likelihood that agricultural pesticides were responsible. But how could these pesticides cause small phallus size and abnormal ovaries?

Lou’s “aha!” moment came when participants of the 1991 Wingspread conference, led by visionary Theo Colborn of the World Wildlife Fund, provided the missing piece of the puzzle. Some pesticides and various other chemicals had been shown to mimic hormones. This could explain the observed effects in his alligator populations; low levels of pesticides were sufficiently estrogenic to induce adverse health effects at critical times during embryonic development, lasting into adulthood. The alligator research led by Lou became one of the most comprehensive and convincing research programs to demonstrate that reproductive problems in wildlife were caused by endocrine-disrupting chemicals. Lou further recognized that these effects could translate to reproductive problems in humans. He showed that alligators act as a sentinel for long-term health effects of environmental exposures, with many parallels to human development and lifespan. There is little doubt that his work on sublethal exposures to environmental contaminants in alligators stimulated research into human reproductive problems. Lou’s work was not without its critics; however, he always held to the mantra of “let the science do the talking.”

Lou had a rare ability to transcend traditional disciplines and unite people for a common cause. His charismatic personality, conviction, and enthusiasm were infectious. He was a dynamic, passionate facilitator and advocate for science education. He felt equally at ease in front of scientists, physicians, students, the general public, and alligators. He inspired a sense of family among those who knew him. His optimism and humor always helped keep things in perspective. His flair for storytelling captivated the many audiences with whom he shared his research message. An avid photographer and aficionado of culture and the arts, his stories would unfold with wonderful images and a delivery that engaged as well as entertained.

Lou’s charisma made him highly sought after by the media. He was an advisor to the Science Communication Network, and his research was featured on programs on Discovery Channel, CBC, PBS, CNN, NHK, and BBC. He was deeply concerned that there are populations of children and wildlife whose health will be compromised because of their exposure to environmental contaminants. With an exceptional ability to communicate and the gravitas of an internationally known researcher, Lou served as a science advisor to many U.S. and foreign agencies and testified before the U.S. Congress and other administrations abroad regarding the impacts of environmental contamination on human and ecosystem health.

Academically, Lou rose to the positions of Distinguished University Professor at the University of Florida and Professor at the Howard Hughes Medical Institute (HHMI) and was a fellow of the American Association for the Advancement of Science. In 2010, Lou joined the Department of Obstetrics and Gynecology at the Medical University of South Carolina as its first alligator biologist, a testament to his ability to translate his work in wildlife to human health. He was also appointed the Center of Economic Excellence (CoEE) Endowed Chair in Marine Genomics with the Hollings Marine Laboratory and became director of the Marine Biomedicine and Environmental Sciences Center and the CoEE Center for Marine Genomics. This was his “dream position,” where he could more effectively tackle how to prevent and treat health problems caused by environmental factors. In 2011 Lou was thrilled at being honored as a Heinz Medal awardee for promoting a healthier environment. He richly deserved this award.

Despite all of Lou’s personal successes, for him science was about people—building relationships, fostering creativity, and providing opportunity. He lived by the motto of a dear mentor, Howard A. Bern: “One’s legacy to science is not the work that you do but the people you leave behind.” Lou was a role model and tireless advocate for mentoring young scientists. He created the HHMI-funded Group-Advantaged Training of Research (GATOR) program to give high school and undergraduate students an opportunity to work alongside faculty and graduate student mentors to learn how science is done and about the importance of academic honesty and research ethics. Young faculty and graduate students received training in how to be effective mentors. Those who have had the privilege of receiving mentorship from Lou are deeply grateful for the life-changing experience.

Lou was an exceptional human being. His death creates a huge hole in many people’s lives and in the field of environmental health. His life, however, provides a wonderful legacy—the people he trained and the passion he instilled in them to continue his quest for harmonizing human activity with environmental and human health.

